# Strategies for optimizing the phase correction algorithms in Nuclear Magnetic Resonance spectroscopy

**DOI:** 10.1186/1475-925X-14-S2-S5

**Published:** 2015-08-13

**Authors:** Franciszek Binczyk, Rafal Tarnawski, Joanna Polanska

**Affiliations:** 1 Silesian University of Technology, Institute of Automatic Control, Data Mining Group,44-100 Gliwice, ul. Akademicka 16, Poland; 2 Maria Sklodowska-Curie Memorial Cancer Centre and Institute of Oncology Gliwice Branch, III Clinic for Radiotherapy, 44-101 Gliwice, ul.Wybrzeze Armii Krajowej 15, Poland

## Abstract

Nuclear Magnetic Resonance (NMR) spectroscopy is a popular medical diagnostic technique. NMR is also the favourite tool of chemists/biochemists to elucidate the molecular structure of small or big molecules; it is also a widely used tool in material science, in food science etc. In the case of medical diagnosis it allows for determining a metabolic composition of analysed tissue which may support the identification of tumour cells. Precession signal, that is a crucial part of MR phenomenon, contains distortions that must be filtered out before signal analysis. One of such distortions is phase error.

Five popular algorithms: Automics, Shanon's entropy minimization, Ernst's method, Dispa and eDispa are presented and discussed. A novel adaptive tuning algorithm for Automics method was developed and numerically optimal solutions to automatic tuning of the other four algorithms were proposed. To validate the performance of the proposed techniques, two experiments were performed - the first one was done with the use of *in silico *generated data. For all presented methods, the fine tuning strategies significantly increased the correction accuracy. The highest improvement was observed for Automics algorithm, independently of noise level, with relative phase error dropping by average from 10.25% to 2.40% for low noise level and from 12.45% to 2.66% for high noise level. The second validation experiment, done with the use of phantom data, confirmed the *in silico *results. The obtained accuracy of the estimation of metabolite concentration was at 99.5%.

**Conclusions:**

The proposed strategies for optimizing the phase correction algorithms significantly improve the accuracy of Nuclear Magnetic Resonance spectroscopy signal analysis.

## Introduction

Magnetic resonance spectroscopy (abr. MRS) is a technique widely used in, among the others, modern oncology to determine metabolic profile of the tissues. It is especially useful to differentiate between healthy tissues and tumours. However the differences in metabolic profiles are in many cases slight, thus signal must be carefully pre-processed in order to accurately estimate amount of metabolites in examined tissues. One of the distortions that affects MR spectrum mostly is phase error. The first simplest mathematical model of phase error was proposed by Ernst [1] where the assumption on linearity of phase error along the spectrum, described by two factors frequency dependent and frequency independent one, was made. Such a model posits the phase error to be mainly a consequence of eddy current induction in the scanner. The extension of linear model is bilinear one, incorporating multiplicatory factor that stands for phase error fraction caused by magnetic field inhomogeneity [2,3]. In contrast to the linear models, higher order models were proposed assuming the nonlinearity of the phase error with respect to the frequency.

While considering MRS in clinical diagnosis, the influence of field inhomogeneity may be neglected due to small spectrum complexity (low dimensionality) and significantly lower field strength (than in chemical measurements), leaving the phase error related to the induction of eddy currents in coils only [4].

The linear model of phase error consists of two parts: zero and first order and it is given by:

$$\text{Δ}\varphi =\varphi_{0}+\frac{\text{k}}{\text{N}}\varphi_{1}$$

Zero order component φ_0 _is representing the offset between absorption and dispersion spectrum while first order component φ_1 _is representing the frequency dependant shift where dependency is modelled by straight line with slope coefficient k/N, where k denotes index of the point in spectrum and N is total number of points.

There are two possible approaches of phasing MR spectra. The first one is manual and requires expert knowledge about zero and first order component. Such a procedure is time consuming and requires an experienced human operator. The drawback of the manual method is the fact that it is hard to estimate the correction effect for the whole spectrum [5]. It is then done for small parts of spectrum and leads to overcorrection of analysed fragments and no correction of other fragments.

The second possible approach that is free of the mentioned drawback is the automatic correction. For such an approach the correction is done accordingly to the linear model, but the quality of spectrum is evaluated and maximized automatically, mostly with the use of optimization techniques. The automatic phase correction algorithms are still less popular than manual approach, which is partially caused by unsatisfactory accuracy of the existing techniques.

The most popular techniques of automatic phasing of MR spectra are: Automics [6], Shanons Entropy minimization [7], Ernst's method [1], Dispa [8] and eDispa [9]. The aim of this paper is to design, implement and verify the automatic tuning strategies for the above mentioned methods that will result in efficiency increase of phase error correction for 1H (Hydrogen isotope Protium) NMR spectra.

## Methods

While looking at the algorithms designed for phase error correction in MR spectra, one can notice two groups of them: model based and model free techniques. In the family of methods that derive direct value of phase error (without a priori assumed error model) the most popular are: [5] which is based on filter diagonalisation method and [10] which performs the phase correction with the use of separately measured water signal. The newest approach of direct phase error correction seems to be the method by [11] requiring the registration of series of spectra.

The five chosen linear model based methods: Automics, Shanon's, Ernst's, Dispa and eDispa, as being the most popular in clinical approaches, were implemented following the description included in the original publications. The mechanism of finding optimal solution was examined for each technique and tuning of algorithms, by means of tuning of parameters or application of efficient numerical solution, was performed.

### Automics

The first analysed algorithm, proposed by [6], is based on estimating linear model parameters φ_0 _and φ_1 _dependent on a phase evaluated at the tails of spectrum. The original method assumes definition of two intervals at each tail. For each pair of intervals a mean phase is calculated. Than having two values of phase: at the beginning and end of the spectrum, phase error is estimated. It is assumed that phase error in these intervals does not differ significantly. The length of the intervals may be understood as a parameter for the algorithm that may be optimized to find the best solution [6].

The developed procedure for interval length estimation starts with the initial interval as a single point and then extends stepwise the interval by other points as long as there is no significant trend in the data within the interval. To verify no trend hypothesis a linear regression model is constructed within each interval and statistical test on signal gradient being equal to zero is applied. For the purpose of this study significance level was set to 5% (α = 0.05). The procedure is repeated for each of two intervals - at the beginning and at the end of the spectrum. If a significant change of signal magnitude (and consequently phase error) is found in one of the intervals the procedure is terminated and the found length is treated as the optimal one. The parameters of phase error model are calculated by solving the set of two equations (for each tail) in the form given by equation below.

$$\text{Δ}\varphi_{j}=\text{atan}\left(\frac{{\text{R}}_{\text{j},2}-{\text{R}}_{\text{j},1}}{{\text{I}}_{\text{j},2}-{\text{I}}_{\text{j},1}}\right)=\varphi_{0+}\left(\frac{\frac{{\text{k}}_{\text{j},1}+{\text{k}}_{\text{j},2}}{2}}{\text{N}}\right)\varphi_{1}$$

Where index j stands for the location of the interval: j = 1 for the interval located at the beginning of the spectrum; while j = 2 for the interval located at the end of the spectrum. R_j,2 _and I_j,2 _are the real and imaginary part of an element at the end of the interval; and R_j,1 _and I_j,1 _are the real and imaginary part of an element at the beginning of the interval, k_j,1 _and k_j,2 _are the indices of the beginning and the end of the j_th _interval, and N is a length of the spectrum.

### Methods based on reformulation to the optimization problem

Three of the above mentioned algorithms might be tuned by application of a properly chosen numerical method for solving their optimization problem.

#### Shanon's entropy minimization

The Shanon's entropy minimization method is based on the assumption that ideal absorption spectrum should be positive. Such a spectrum has smaller Shanon's entropy than spectrum that contains points that are both: positive and negative [7]. The problem for this method is to find set of parameters φ_0_, φ_1 _for which the spectrum phased with linear model has the smallest error of correction. The minimization problem is given by equation.

$${\text{min}}_{\varphi_{0},\varphi_{1}}H={\text{min}}_{\varphi_{0},\varphi_{1}}\left(-\sum_{\text{k}=1}^{\text{N}}{\text{S}}_{\text{A}}\left(k,\varphi_{0},\varphi_{1}\right)\cdot \text{ln}\left({\text{S}}_{\text{A}}\left(k,\varphi_{0},\varphi_{1}\right)\right)+\text{P}\right)$$

where H is the Shannon entropy of given spectrum, S_A_(k,...) is a magnitude of the absorption spectrum at k_th _data point and P is a penalty factor.

#### Ernst

Ernst method is based on the axiom that the integral of single line (peak) dispersion spectrum should be equal to zero. Since the spectrum is a composition of peaks it is clear that for no phase error the dispersion integral of whole spectrum should be zero [1]. Because of the noise it is rarely to be true. In the Ernst method the optimization problem is to find parameters of linear model for which the dispersion integral will be minimal.

$${\text{min}}_{\varphi_{0},\varphi_{1}}I={\text{min}}_{\varphi_{0},\varphi_{1}}\overset{\text{b}}{\underset{\text{a}}{\int }}{\text{S}}_{\text{D}}\left(x,\varphi_{0},\varphi_{1}\right)dx$$

where I is an integral value, S_D _is a magnitude of the dispersion spectrum; a and b are the integration limits equivalent to the minimum and maximum values on the frequency [Hz] or [ppm] scale of the spectrum.

#### eDispa

In eDispa method authors use linear model as well and perform the two-step quality calculation [9]. The first step is based on calculation of defined η -functional of the form given below.

$${\text{max}}_{\varphi_{0},\varphi_{1}}\eta \left(\varphi_{0},\varphi_{1}\right)={\text{max}}_{\varphi_{0},\varphi_{1}}2\pi {\left(\frac{\text{Q}\left(\varphi_{0},\varphi_{1}\right)-\text{min Q}\left(\varphi_{0},\varphi_{1}\right)}{\text{max Q}\left(\varphi_{0},\varphi_{1}\right)-\text{min}\text{Q}\left(\varphi_{0},\varphi_{1}\right)}\right)}^{4}$$

Q is a functional defined as:

$$\text{Q}\left(\varphi_{0},\varphi_{1}\right)=\overset{\text{N}}{\underset{\text{k}=1}{\sum }}{\left({\text{S}}_{\text{A}}\left(\text{k},\varphi_{0},\varphi_{1}\right)\right)}^{2}\text{exp}\left(\frac{-2\cdot \left(2\text{k}-\text{N}\right)}{\text{N}}\right)$$

where S_A _denotes the magnitude of the absorption spectrum, k is the index of data point, N is a length of the spectrum.

### Solutions to the optimization problems

The solution to three above mentioned algorithms could be found with the use of classical optimization algorithms. In case of Shanon's entropy minimization and eDispa problem the Nelder-Mead algorithm can be applied [12]. As for Ernst's method the problem is more complex, and it may be solved with the use of integral global optimization [13].

One of the crucial steps during the Nelder-Mead optimization, due to the strong nonlinearity of the optimized functions, is setting of parameter initial values. To improve accuracy of tuned algorithms and to make whole optimization process faster the procedure for setting of initial conditions has been proposed. It is based on observation that water peak is located in the middle of spectrum and it is with no doubt the peak of maximal amplitude both in absorption and magnitude spectrum. As described in previous paragraph, a phase angle between absorption and dispersion part measured at peak maximum should be equal 0. If it is not, the measured value is a rough estimate of phase error at the half of spectrum:

$$\text{Δ}\varphi^{0}=\varphi \left({\text{S}}_{{\text{k}}_{\text{max}}}\right)$$

where Δφ^0 ^is an initial estimate of the phase error, and S stands for the MR spectrum, index k_max _denotes the spectrum data point with maximum of magnitude spectrum.

**Figure 1:**
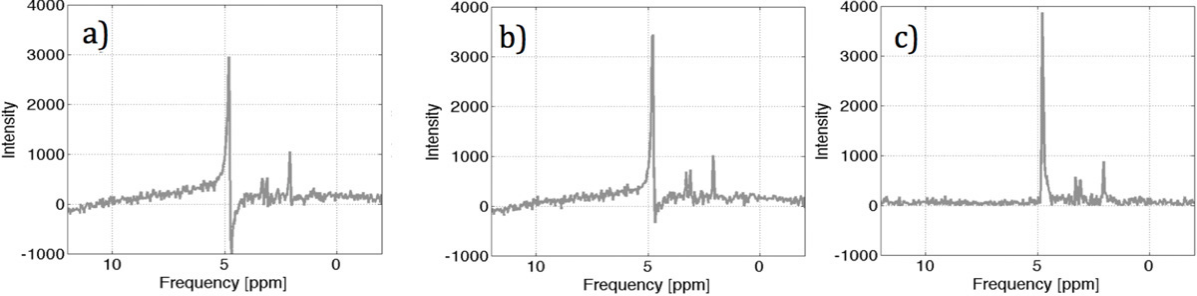
**a) Spectrum before phase correction, b) spectrum after correction with random initial condition, c) spectrum corrected with proposed initial condition**.

Knowing the value of Δφ^0 ^it is easy to estimate φ_0 _and φ_1 _just using the equation for linear phase error model and additional assumption that ratio of phase error components is equal ¼. This value is empirical and it was chosen after set of experiments done on clinical spectra. Example of effectiveness of the proposed initial condition is shown in Figure 1.

The proposed initial condition may be used even when water signal is partially suppressed during measurement procedure. When water signal is not present in the data (full water suppression) the maximum of signal may be used, however it is definitely not as good as water peak.

#### Dispa

Dispa method is based on the assumption that phase at the maximum of the peak should, in an ideal case, be equal 0 [8]. Assuming the linear model of Δφ it is then easy to estimate φ_0 _and φ_1 _with use of just two neighbouring peaks. It was noticed that such approach might lead to wrong estimates because of noise presence and its influence on maximal point of peak. An idea for Dispa method is to evaluate phase value at max points of all significant peaks and then estimate Δφ model with use of linear regression model.

### Quality criterion

**Figure 2:**
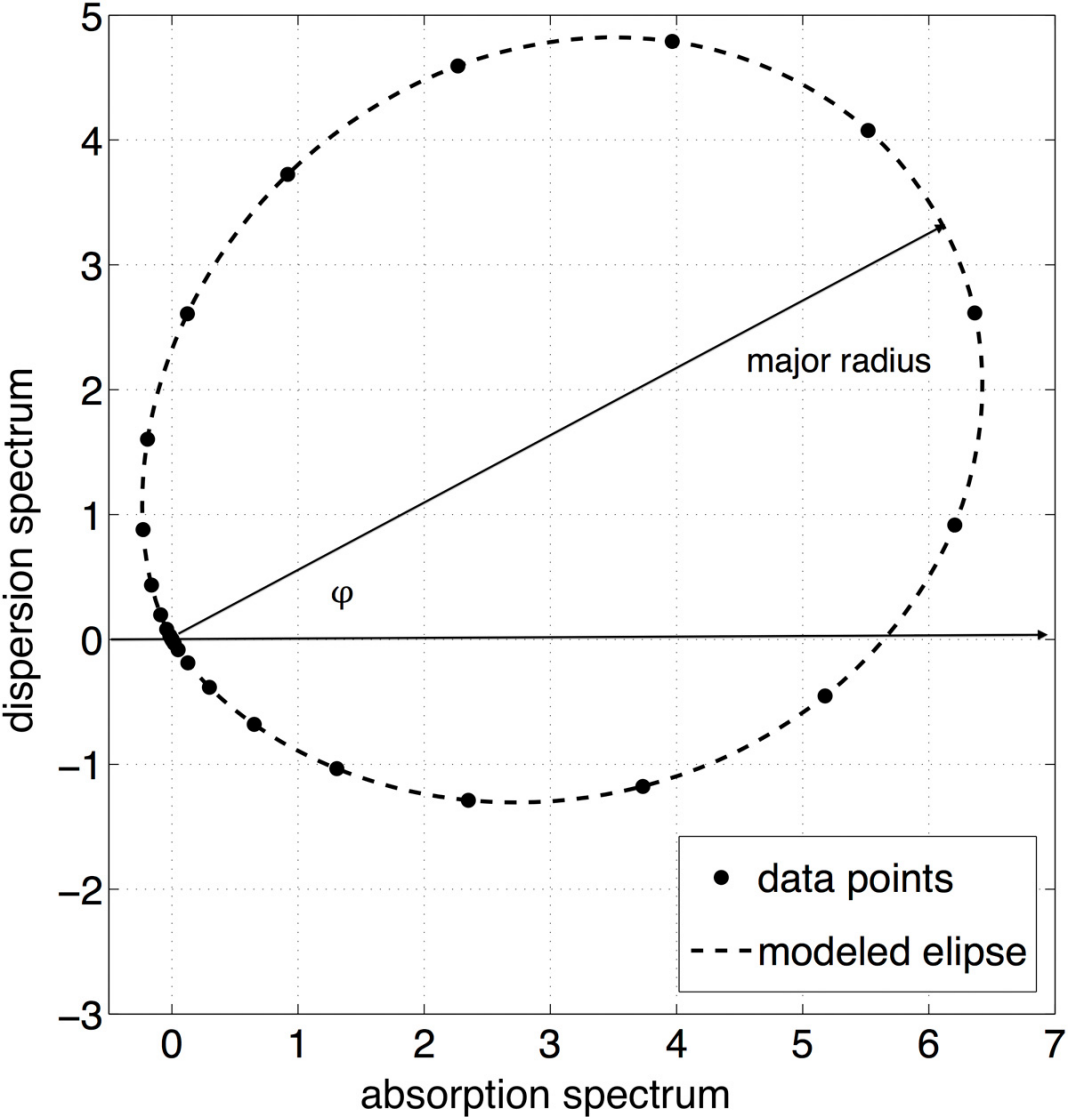
**Visualization of idea behind assumed quality criterion **[15].

In order to properly estimate value of phase error that remains in the data after phase correction, a quality criterion was proposed. The assumed criterion uses the phase plot (relation between dispersion and absorption spectrum), obtained for last significant peak in the analysed spectrum. Because of signal sampling a peak and consequently phase plot is not a continuous line but a set of points. Because the criterion uses major radius of phase plot, an estimates of ellipse parameters are obtained from the data points. Having ellipse equation it is then easy to derive the equation for its major and minor radius. The assumption is that in case of no phase error, major radius of an ellipse phase plot should lay exactly on the real axis. The remaining phase error is the angle between real axis of the phase plane and major radius of an ellipse phase plot. The idea of the assumed quality criterion is shown in Figure 2.

### Data

To verify the quality of spectra phasing two data sets were collected. The first one consists of numerically simulated spectra (named as *synthetic data)*, while the second data set consists of 27 measurements obtained for a brain phantom that contained: 5 mM of Lactates at 0.5 ppm, 12.5 mM of *N*-acetylaspartate (abr. NAA) at 2.0 ppm, 10 mM of Creatine at 3.0 ppm, 3 mM of Choline at 3.2 ppm and 7.5 mM of myo-Inositol located at 4.6 ppm. The data were measured with the use of Philips Achieva scanner of 1.5 T magnetic field induction. The echo and repetition time were equal to 35 and 1500 ms. Every spectrum was averaged over 128 replicates. The number of points was equal to 1024 and the sequence type was PRESS.

To obtain the synthetic data set being similar in structure to the brain data, randomly chosen single spectrum was taken from phantom data and it was manually phased by human expert. Then the absorption spectrum was extracted and pre-processed to filter out noise and baseline. The smoothed signal was used as the reference for synthetic signal generator.

Single synthetic spectrum was generated with the use of the following procedure:

1) To ensure signal complexity similar to the clinical spectra, additional peaks together with additive noise were randomly added to the reference signal in frequency domain.

2) The dispersion spectrum was reconstructed with the use Hilbert transformation.

3) Signal was disturbed with additive phase error of parameters: φ_0 _= {2.5, 5.0, 7.5, 10.0, 12.5} degrees and φ_1 _= {2.5, 5.0, 7.5, 10.0, 12.5} degrees.

4) Additionally, signal was disturbed by additive noise with SNR equal to: 30.75 dB (named *low noise*) and 8.52 dB (named *high noise*).

**Figure 3:**
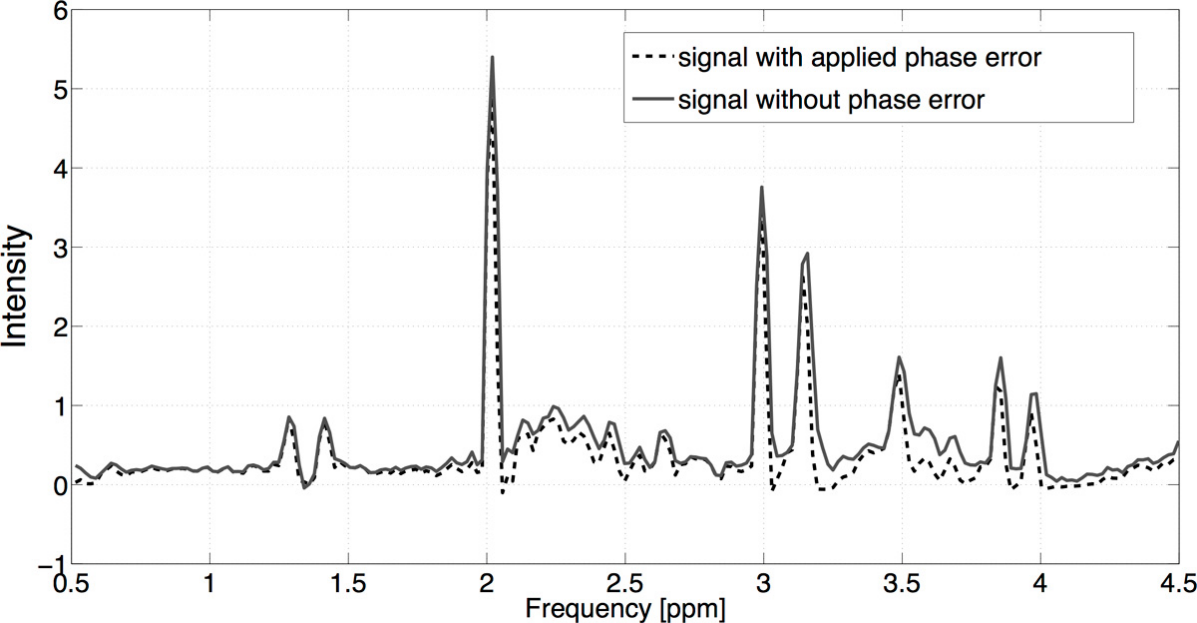
**Exemplary spectrum obtained with the addition of the phase error equal to 10 degrees and low level noise**. [15]

Since the noise component was purely random each combination of φ_0 _and φ_1 _was repeated 50 times. In total 1250 simulations were performed. Exemplary simulated spectrum is presented in Figure 3.

### Evaluation

To evaluate the efficiency of the proposed strategies for algorithm tuning, every spectrum was corrected twice, by original and tuned algorithm. The residual post correction error was calculated following the procedure described in Quality criterion section. The error residuum expressed as a percentage of the applied phase distortion value was named a relative error.

**Figure 4:**
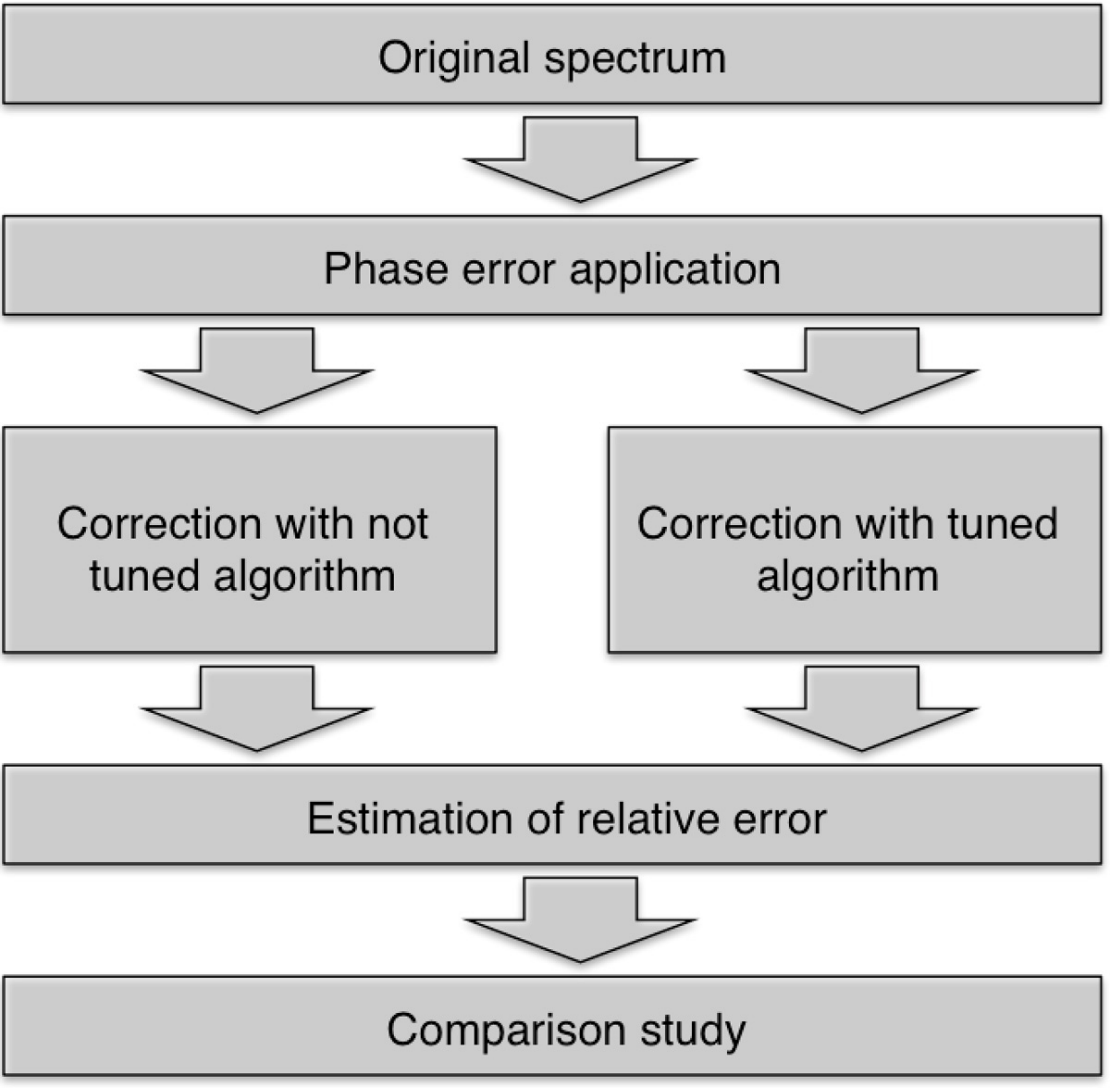
**Proposed experiment scheme**. Both tuned and not tuned methods are examined [15].

The block diagram describing performed comparison study is shown in Figure 4.

## Results

### Experiment I - synthetic data

The experiment was performed for 9 values of Δφ = {5.0, 7.5, 10.0, 12.5, 15.0, 17.5, 20.0, 22.5 and 25.0} degrees (obtained for different combination of φ_0 _and φ_1 _in a range: 2.5, 5, 7.5, 10 and 12.5 degrees each). Each combination of Δφ was distorted with an additive noise of 30.75 dB (low) or 8.52 dB (high) and repeated 50 times what results in 1250 simulations in total. The correction was applied to every generated spectrum in both manners: with the use of original algorithm and with applied tuning routines. The relative error was calculated, and the results were grouped with respect to the total Δφ value. For each group mean value, standard deviation and coefficient of variation CV were calculated.

#### Low level of additive noise

Basing on the above results it may be noticed that proposed tuning routines improve phase correction quality for each of the analysed algorithm. The highest increase was observed for algorithm Automics and the lowest increase was observed for Ernst algorithm. By looking at a mean value of relative error it may be concluded that with the increase of Δφ the remaining phase error after the correction increases in both cases (before and after tuning). By looking at descriptive statistics, for correction with the use of tuned algorithms the dispersion of results among spectra with different noise is much lower for Automics, Shanon's and Ernst but remains at the same level for Dispa and eDispa.

#### High level of additive noise

The second part of the synthetic data experiment differs from the first by a much larger additive noise that was applied to all generated spectra. As previously a huge improvement in results obtained for with tuning was noticed. For all five algorithms the values of relative phase error are worse while compared to the low noise level results. The best results were obtained for Automics algorithm. It was also observed that addition of higher-level noise increases the dispersion of spectra generated for same combination of φ_0 _and φ_1 _values. With low noise this value was equal to ~1% for all methods without tuning, while by increase of the noise level it doubles. By application of tuning it was possible to decrease the dispersion to about 1%.

### Experiment II - brain phantom data analysis

In the second part of validation procedure a data obtained on brain phantom was analysed. The number of processed spectra was equal to 27. All signals were collected at different time frames with use of Philips Achieva (1.5 T) with parameters: echo time = 35 ms, repetition time = 1500 ms, number of averages = 128, number of points = 1024 and the sequence type PRESS. Thus it was assumed that the distortions such as phase errors or noise would be different from spectrum to spectrum. Following the results of the analysis performed on synthetic data, each phantom spectrum phase was corrected with the use of Automics algorithm only. The correction was performed twice: with and without parameter tuning. The spectra were then decomposed into Gaussian Mixture Model (abr. GMM) and the concentrations of metabolites were calculated [14]. The obtained estimates of concentrations are presented in the form of boxplots in Figure 7 and their descriptive statistics are included in table 5.

**Figure 5:**
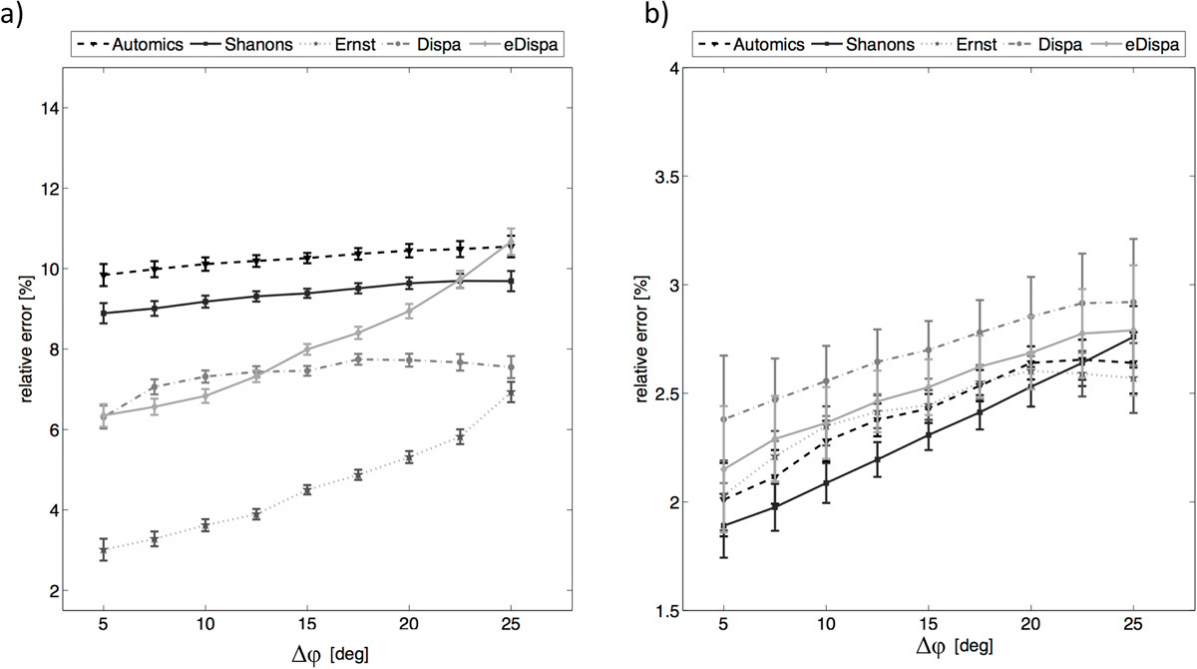
**The relative phase error [%] and its confidence intervals obtained for phase correction algorithms a) before and b) after the application of tuning procedures - low noise**.

**Figure 6:**
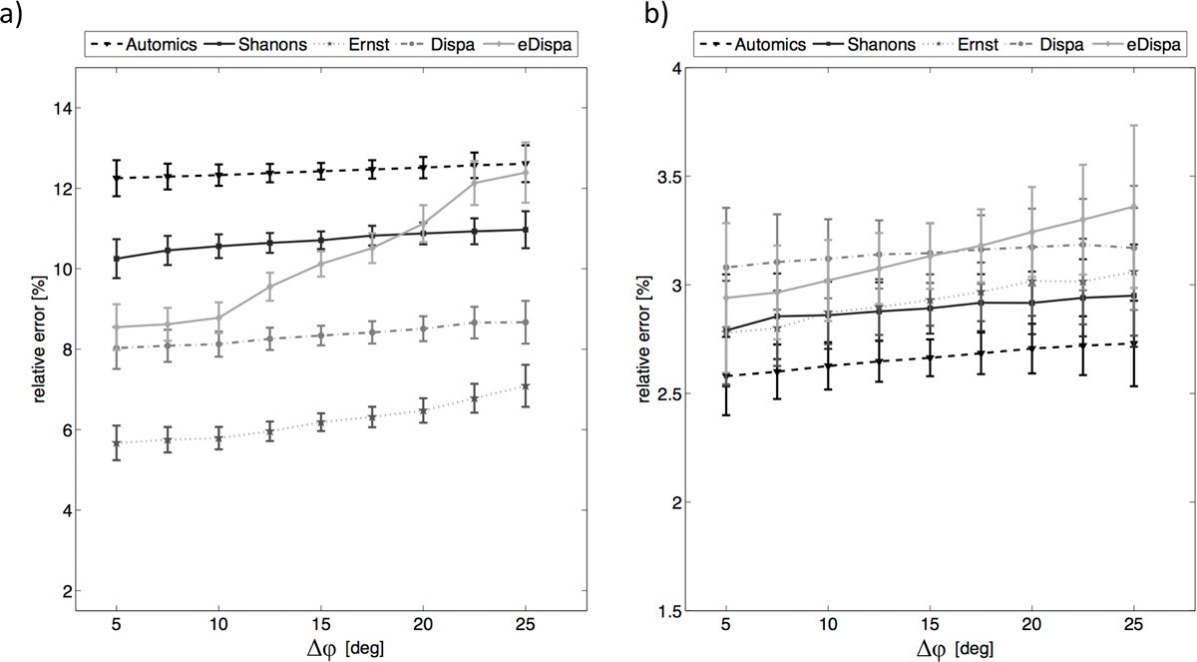
**The relative phase error [%] and its confidence intervals obtained for phase correction algorithms a) before and b) after the application of tuning procedures - high noise**.

**Figure 7:**
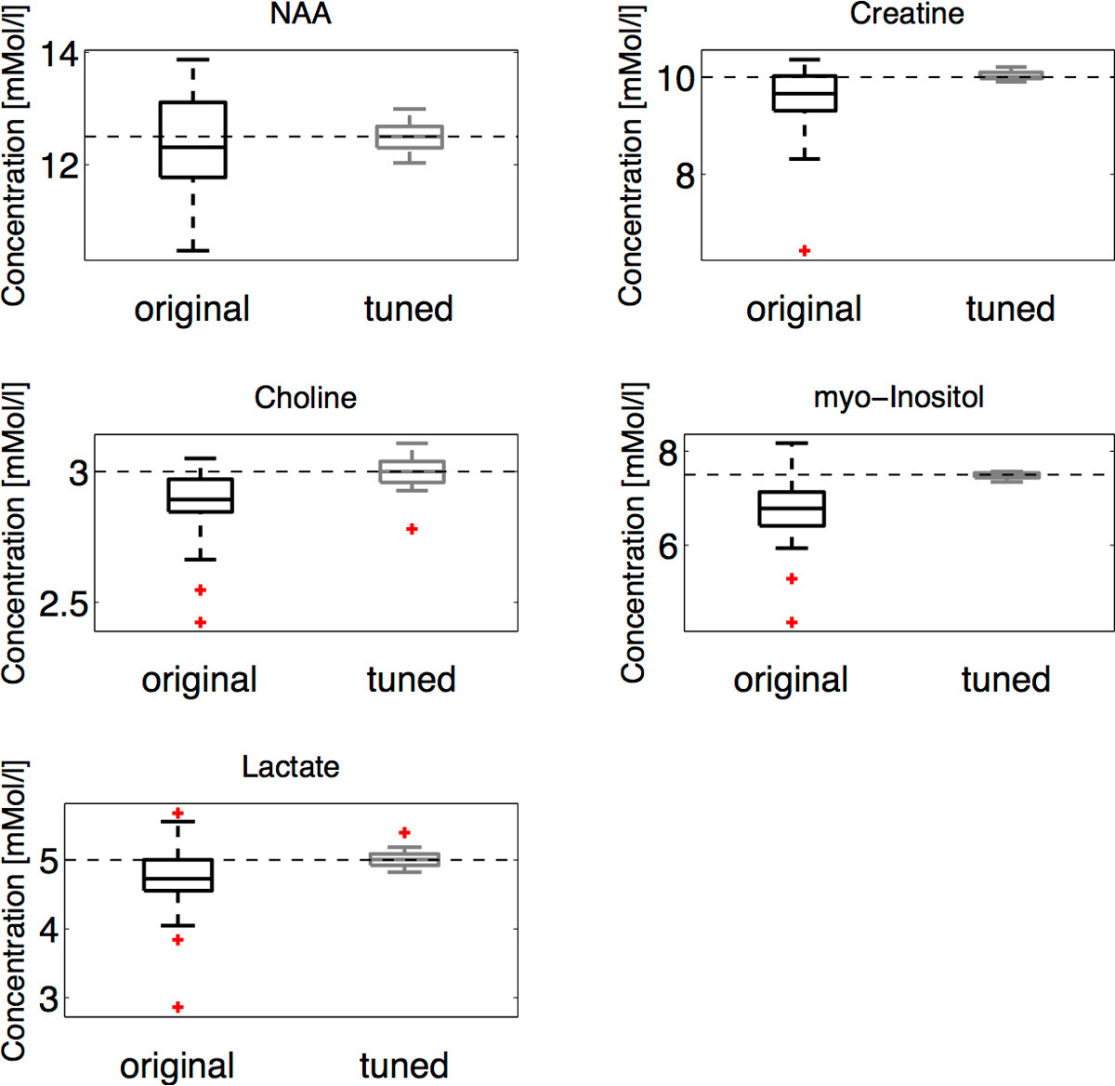
**Distributions of metabolite concentration calculated for 27 spectra obtained on brain phantom**. For each spectrum two experiments were performed: with phase correction by original Automics algorithm (original) and second by tuned Automics (tuned). Boxplots represent median and upper and lower quartiles of distribution, Tukey's criterion was used for outlier detection (marked as dots). For each metabolite desired value is indicated by dotted line.

**Table 1:** The statistics of location (mean) and dispersion (standard deviation and coefficient of variation) for relative phase error [%] obtained for phase correction algorithms before the application of tuning procedures - low noise.

**Δ**φ	Automics			Shanon's			Ernst			Dispa			eDispa		
	
	$\bar{x}$	***s***	CV [%]	$\bar{x}$	***s***	CV [%]	$\bar{x}$	***s***	CV [%]	$\bar{x}$	***s***	CV [%]	$\bar{x}$	***s***	CV [%]
5.00	9.84	0.99	10.06	8.89	0.91	10.24	3.01	0.98	32.56	6.31	1.03	16.32	6.35	1.01	15.91

7.50	9.99	1.02	10.22	9.01	0.94	10.38	3.28	0.94	28.51	7.06	0.96	13.53	6.57	1.03	15.61

10.00	10.11	1.04	10.32	9.18	0.93	10.17	3.62	0.93	25.78	7.32	0.94	12.89	6.83	1.06	15.56

12.50	10.19	1.05	10.28	9.31	0.92	9.91	3.89	0.94	24.08	7.44	0.98	13.21	7.32	1.06	14.51

15.00	10.26	1.03	10.06	9.39	0.92	9.80	4.50	0.93	20.71	7.46	0.98	13.19	7.99	1.09	13.59

17.50	10.37	1.04	10.06	9.51	0.92	9.70	4.87	0.92	18.88	7.75	0.97	12.56	8.40	1.11	13.15

20.00	10.45	1.04	9.96	9.64	0.91	9.44	5.31	0.93	17.50	7.72	1,00	12.99	8.94	1.13	12.60

22.50	10.49	1.02	9.68	9.70	0.9	9.28	5.82	0.93	15.98	7.67	1.05	13.62	9.73	1.12	11.51

25.00	10.55	0.97	9.19	9.69	0.91	9.39	6.93	0.91	13.13	7.55	0.99	13.11	10.67	1.18	11.06

**Table 2:** The statistics of location (mean) and dispersion (standard deviation and coefficient of variation) for relative phase error [%] obtained for phase correction algorithms with applied tuning - low noise.

**Δ**φ	Automics			Shanon's			Ernst			Dispa			eDispa		
	
	$\bar{x}$	***s***	CV [%]	$\bar{x}$	***s***	CV [%]	$\bar{x}$	***s***	CV [%]	$\bar{x}$	***s***	CV [%]	$\bar{x}$	***s***	CV [%]
5.00	2.01	0.61	30.35	1.89	0.53	28.04	2.03	0.58	28.57	2.38	1.06	44.54	2.15	1.05	48.84

7.50	2.12	0.63	29.79	1.98	0.55	27.85	2.21	0.60	26.92	2.47	0.97	39.27	2.29	1.01	44.10

10.00	2.28	0.58	25.58	2.09	0.57	27.48	2.35	0.56	23.97	2.56	1.01	39.50	2.36	1.03	43.58

12.50	2.38	0.55	22.92	2.20	0.58	26.20	2.42	0.55	22.57	2.65	1.08	40.74	2.46	1.03	41.62

15.00	2.43	0.54	22.14	2.31	0.56	24.35	2.45	0.55	22.57	2.70	1.07	39.70	2.53	1.04	40.98

17.50	2.54	0.52	20.51	2.41	0.57	23.63	2.55	0.55	21.37	2.78	1.08	38.67	2.62	1.03	39.37

20.00	2.64	0.48	18.06	2.53	0.57	22.53	2.60	0.52	20.10	2.85	1.14	39.95	2.69	1.05	39.21

22.50	2.66	0.47	17.70	2.64	0.55	20.64	2.59	0.54	20.66	2.92	1.17	39.97	2.78	1.05	37.66

25.00	2.64	0.51	19.32	2.76	0.51	18.48	2.57	0.58	22.57	2.92	1.05	35.96	2.79	1.08	38.71

**Table 3:** The statistics of location (mean) and dispersion (standard deviation and coefficient of variation) for relative phase error [%] obtained for phase correction algorithms before the application of tuning procedures - high noise.

**Δ**φ	Automics			Shanon's			Ernst			Dispa			eDispa		
	
	$\bar{x}$	***s***	CV [%]	$\bar{x}$	***s***	CV [%]	$\bar{x}$	***s***	CV [%]	$\bar{x}$	***s***	CV [%]	$\bar{x}$	***s***	CV [%]
5.00	12.25	1.61	13.14	10.25	1.75	17.07	5.67	1.55	27.34	8.03	1.88	23.41	8.55	2.05	23.98

7.50	12.29	1.64	13.30	10.46	1.86	17.74	5.75	1.62	28.09	8.09	2.04	25.17	8.62	2.09	24.19

10.00	12.33	1.66	13.47	10.56	1.83	17.36	5.79	1.75	30.22	8.13	1.95	24.04	8.78	2.39	27.26

12.50	12.38	1.64	13.27	10.64	1.78	16.75	5.96	1.76	29.45	8.26	1.99	24.10	9.55	2.52	26.33

15.00	12.42	1.64	13.23	10.71	1.76	16.42	6.19	1.78	28.81	8.34	1.98	23.69	10.12	2.55	25.24

17.50	12.47	1.65	13.25	10.82	1.76	16.26	6.32	1.84	29.14	8.42	2.00	23.76	10.51	2.68	25.49

20.00	12.51	1.65	13.19	10.88	1.69	15.57	6.48	1.89	29.23	8.51	1.94	22.76	11.12	2.87	25.78

22.50	12.57	1.62	12.89	10.93	1.65	15.05	6.78	1.83	26.99	8.66	2.01	23.21	12.13	2.80	23.04

25.00	12.61	1.65	13.08	10.97	1.66	15.13	7.09	1.89	26.66	8.67	1.92	22.15	12.39	2.71	21.87

**Table 4:** The statistics of location (mean) and dispersion (standard deviation and coefficient of variation) for relative phase error [%] obtained for phase correction algorithms with applied tuning - high noise.

**Δ**φ	Automics			Shanon's			Ernst			Dispa			eDispa		
	
	$\bar{x}$	***s***	CV [%]	$\bar{x}$	***s***	CV [%]	$\bar{x}$	***s***	CV [%]	$\bar{x}$	***s***	CV [%]	$\bar{x}$	***s***	CV [%]
5.00	2.58	0.65	25.19	2.79	0.93	33.33	2.78	0.86	30.94	3.08	0.99	32.14	2.94	1.24	42.18

7.50	2.60	0.64	24.62	2.86	1.01	35.20	2.80	0.89	31.61	3.11	1.12	36.07	2.97	1.10	37.10

10.00	2.63	0.68	25.89	2.86	0.97	33.80	2.87	0.92	31.90	3.12	1.14	36.43	3.02	1.17	38.63

12.50	2.65	0.68	25.59	2.88	0.97	33.54	2.90	0.93	31.92	3.14	1.13	36.07	3.08	1.18	38.46

15.00	2.66	0.68	25.68	2.89	0.94	32.57	2.93	0.95	32.49	3.15	1.11	35.35	3.13	1.22	38.83

17.50	2.69	0.69	25.79	2.92	0.95	32.39	2.97	0.98	32.86	3.16	1.14	36.13	3.18	1.21	38.05

20.00	2.71	0.71	26.35	2.92	0.90	30.86	3.02	1.00	33.04	3.17	1.11	34.87	3.24	1.29	39.88

22.50	2.72	0.69	25.37	2.94	0.91	30.78	3.02	1.01	33.33	3.19	1.08	33.75	3.30	1.29	39.09

25.00	2.73	0.71	26.01	2.95	0.85	28.81	3.06	1.06	34.64	3.17	1.03	32.49	3.36	1.35	40.18

**Table 5:** Results of the analysis of 27 brain phantom spectra obtained for two different phase correction algorithms: original Automics and Automics with proposed tunning applied.

Metabolite	not tuned				tuned				paired t-test p-values
	
	$\bar{x}$	s	95% CI	CV [%]	$\bar{x}$	s	95% CI	CV [%]	
NAA	12.32	0.87	(12.25; 12.39)	7.48	12.51	0.24	(12.33; 12.69)	2.33	0.0023

Creatine	9.51	0.79	(9.45; 9.57)	8.36	10.04	0.09	(9.95; 10.13)	1.15	0.0048

Choline	2.87	0.14	(2.86; 2.88)	4.93	3.00	0.06	(2.85; 3.14)	1.89	0.0023

myo-Inositol	6.67	0.73	(6.61; 6.73)	10.81	7.49	0.06	(7.38; 7.59)	1.36	0.0048

Lactate	4.72	0.58	(4.67; 4.76)	12.07	5.02	0.12	(4.86; 5.17)	2.00	0.0023

The presented descriptive statistics are: sample mean , sample standard deviation (s), 95% confidence interval for population mean value (95%CI) and coefficient of variation (CV). Right column presents paired t test p-value resulting from testing the hypothesis on no accuracy improvement by algorithm tuning.

One can conclude that the tuning routine applied to the Automics algorithms improves the results of phase correction giving more accurate estimates of metabolite concentrations. In comparison to the algorithm before tuning the increase is significant for each analysed metabolite. After application of tuning procedure the maximum difference between estimated mean and the true values of metabolite concentration is 0.4% (10.04 vs. 10.00 mM for Creatine and 5.02 vs. 5.0 for Lactate). Estimated mean concentration of Choline is exactly at desired value of 3.0 mM. By looking at the values of standard deviation and coefficient of variation it may be noticed that for all metabolites dispersion of results among 27 spectra is smaller while compared to not tuned version (highest increase for myo-Inositol: standard deviation for not tuned Automics was 0.73 while 0.06 for tuned algorithm).

## Discussion

The *in silico *experiment was performed to verify effectiveness of the proposed tuning for five popular phase correction algorithms. Parameter tuning increases the correction efficiency by at least 4%. The higher impact of tuning algorithm is observed for higher phase errors. The highest impact was observed for Automics algorithm for which modification was the most complex. The adaptive definition of interval length is more efficient than the fixed length option. Additionally, it minimizes the risk that interval contains points that significantly differ in magnitude and phase error. For the group of methods based on the reformulation to the optimization problem it was observed that implementation of efficient simplex algorithm increased accuracy of all three of them. It is also a result of efficient setting of the initial condition. The high (around 5%) improvement was observed for the tuned version of Dispa algorithm. It is a result of accurate estimation of Δφ parameters with the use of all peaks not just selected two. Because of the noise presented in the data, the position of maximal point in the peak is shifted. If the observed maximum is not a true maximum, the phase evaluated at that point is also wrong. For lower noise the correction accuracy is slightly better but that was expected.

In the analysis of clinical phantom data it was proven that tuned algorithm outperforms not tuned version. Only the Automics algorithm was used for phase correction, as it was demonstrated to be the best performing during the synthetic data analysis. Its original version gives results that are satisfactory however we have proven that tuning may increase accuracy and may decrease the dispersion of metabolite concentration estimates among the spectra.

## Conclusions

The proposed tuning routines significantly increase the accuracy of phase error correction for all examined algorithms: Automics, Shanon's entropy minimization, Ernst's, eDispa and Dispa. To understand the importance of proper spectrum phasing two-step validation experiment was performed. The first one was based on the analysis of spectra with known phase error disturbed by additional random noise (synthetic data), while the second validation experiment was performed on spectra with unknown phase error but known original concentration of metabolites. Both validation experiments showed that tuning routines increase the accuracy. The second, phantom based validation experiment has shown that phase error correction the crucial role in determining the metabolite concentration and may lead to more accurate clinical diagnosis.

## Competing interests

We hereby confirm that the authors of this manuscript do not have any competing financial, professional or personal interests.

## Authors' contributions

We hereby declare that FB contributed in the developing methodology for data analysis and performed the numerical experiments. JP contributed in designing the experiment, developing methodology for data analysis and analysed and discussed results. RT contributed in the NMR methodology part.

## Declarations

Publication of this article was supported by NCN grant Harmonia 4 no. DEC-2013/08/M/ST6/00924. Calculations were carried out using infrastructure of GeCONiI (POIG.02.03.01-24-099/13).

This article has been published as part of *BioMedical Engineering OnLine *Volume 14 Supplement 2, 2015: Select articles from the 2nd International Work-Conference on Bioinformatics and Biomedical Engineering (IWBBIO 2014). The full contents of the supplement are available online at http://www.biomedical-engineering-online.com/supplements/14/S2.
